# Nucleant layer effect on nanocolumnar ZnO films grown by electrodeposition

**DOI:** 10.1186/1556-276X-8-135

**Published:** 2013-03-23

**Authors:** Maria D Reyes Tolosa, Laura C Damonte, Hicham Brine, Henk J Bolink, María A Hernández-Fenollosa

**Affiliations:** 1 Instituto de Tecnología de Materiales, Universitat Politècnica de València, Camino de Vera s/n, Valencia, 46022, Spain; 2 Departamento de Física, Facultad de Ciencias Exactas, Universidad Nacional de La Plata-IFLP, CCT, CONICET, C.C.67, La Plata, 1900, Argentina; 3 Instituto de Ciencia Molecular, Universidad de Valencia, C/ Catedrático J. Beltrán 2, Paterna, ES-46980, Spain

**Keywords:** Spin coating, Electrodeposition, ZnO films, DC magnetron sputtering

## Abstract

Different ZnO nanostructured films were electrochemically grown, using an aqueous solution based on ZnCl_2_, on three types of transparent conductive oxides grow on commercial ITO (In_2_O_3_:Sn)-covered glass substrates: (1) ZnO prepared by spin coating, (2) ZnO prepared by direct current magnetron sputtering, and (3) commercial ITO-covered glass substrates. Although thin, these primary oxide layers play an important role on the properties of the nanostructured films grown on top of them. Additionally, these primary oxide layers prevent direct hole combination when used in optoelectronic devices. Structural and optical characterizations were carried out by scanning electron microscopy, atomic force microscopy, and optical transmission spectroscopy. We show that the properties of the ZnO nanostructured films depend strongly on the type of primary oxide-covered substrate used. Previous studies on different electrodeposition methods for nucleation and growth are considered in the final discussion.

## Background

Nanostructured ZnO thin films required a controlled fabrication process for many applications based on semiconductor devices. ZnO thin films have been prepared by a wide variety of techniques such as pulsed laser deposition [1,2], sputtering [3,4], and electrodeposition with or without templates [5-8]. In particular, the electrodeposition technique has advantages over other processes due to its simplicity, low equipment cost, and the possibility of obtaining large-area thin films. Also, electrodeposition is an efficient and reliable technique for preparing ZnO nanocrystallites [9], nanowires [10,11], and nanorods [5,12]. One of the key elements to achieve high efficiency on nanostructured heterojunctions is the control on density, morphology, and crystallinity during growth [13]. The resulting film surface morphology depends on a variety of parameters, like initial solution, ion concentration, bath temperature, etc. [14]. To improve nanostructure morphology of electrodeposited films, post-heat treatments are usually applied [15]. In this sense, the evolution of optical and morphological properties with the annealing temperature for ZnO electrodeposited films on FTO was analyzed in a previous work [16]. Recently, it has been found that the presence of a seed layer plays an important role in the properties of the nanostructured films grown on top of them by different methods such as hydrothermal synthesis [17-19]. This seed layer guaranteed a well-defined orientation and alignment of the grown nanostructures, as well as optical property improvements due to their very low roughness and small particle size. Additionally, these primary oxide layers prevent direct hole combination when used in optoelectronic devices [20].

In this work, the influence of different seed layers on the structural and optical properties of electrodeposited ZnO nanorods is analyzed. The transparent conductive oxide layer as seed layer was prepared by three different methods: (1) spin-coated ZnO, (2) direct current (DC) magnetron sputtered ZnO, and (3) commercial ITO (In_2_O_3_:Sn)-covered glass substrates.

The ZnO growth process was also varied, taking into account previous studies on different electrodeposition procedures for nucleation and growth [5,13]. Potentiostatic, galvanostatic, and pulsed-current electrochemical deposition methods were applied for each seed layer, analyzing their influence on the general properties of the obtained nanostructure.

We have analyzed morphological and structural properties by scanning electron microscopy (SEM) and atomic force microscopy (AFM), and optical properties by transmission spectra. Optical bandgap was determined by Tauc's plot.

## Methods

### ZnO spin coated on ITO

A ZnO nucleant layer of 20-nm thickness and wurtzite crystalline structure was obtained by spin-coating technique. The substrates were 3 × 3-cm^2^ ITO (indium tin oxide)-sputtered glass (resistivity at room temperature, 15 Ω/cm^2^) from Asahi Glass Company (Tokyo, Japan). The solution used was a reagent-grade (RG) zinc acetate [Zn(CH_3_COO_2_) · 2H_2_O] dissolved in RG methanol in a 0.02-mol/l solution.

Previously, the substrate was cleaned with neutral soap for 10 min in ultrasonic bath, 10 min in distilled water, 10 min in isopropanol, and finally dried with N_2_. The spin-coating process was done dropping 0.2 ml of solution on the cleaned substrate and rotating it at 3,000 rpm. Then, heat treatment at 80°C was necessary to evaporate the organic component from the layer.

### ZnO sputtered on ITO

The second ZnO nucleant layer was prepared by DC sputtering process on the same ITO substrate described in the section ‘ZnO spin coated on ITO’ from a ZnO target of 99.999% purity. A homemade sputtering system with a power of 100 W, 2 × 10^−2^ mbar of Ar pressure, and a substrate temperature of 300°C was used. The layer obtained has 60-nm thickness and a stable wurtzite crystalline structure.

### Growth of ZnO nanorods on three different substrates

ZnO nanorods were obtained by electrochemistry technique in a classical three-electrode electrochemical cell, with the spin-coated ZnO films, sputtered ZnO films, or ITO substrates as the working electrode. A platinum sheet and Ag/AgCl (3 M KCl) were used as auxiliary and reference electrodes, respectively. The electrolyte used was 5 × 10^−3^ M ZnCl_2_ (RG) and 0.1 M KCl (RG) solution with O_2_ saturation working at 70°C during the whole electrodeposition process. The experiments were carried out in an Autolab PGSTAT302N potentiostat (Metrohm, Utrecht, The Netherlands) with an ADC 10M card for ultrafast measurement acquisition (one sample every 10 ns). The electrochemical experiments were performed potentiostatically for 10 min, galvanostatically for 10 min, and by pulsed current at a frequency of 0.5 Hz for 20 min, for each of the substrates.

**Figure 1:**
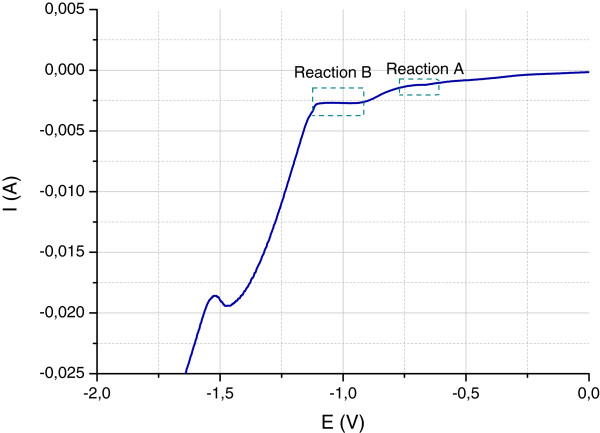
**Linear voltammetry curve.** ZnCl_2_ 5 × 10^−3^ M and 0.1 M KCl at 70°C on ITO substrate at 0.1 V/s.

The optimal potential for each substrate was chosen by means of a cyclic voltammetry curve with the same variable process of 0.1 V/s. As an example, a current–voltage study performed under these conditions for the ITO substrate is shown in Figure 1. Two different stages on the deposition branches can be distinguished, corresponding to the dominant reactions:

Reaction A: Zn^+2^ + 0.5 O_2_ + H_2_O→ 2*e*^−^ + Zn(OH)_n_

Reaction B: Zn^+2^ + 0.5 O_2_→ 2*e*^−^ + ZnO

Table 1 shows the electrochemical parameters applied for the potentiostatic, galvanostatic, and pulsed-current growth of the ZnO process for each nucleant layer.

**Table 1:** Electrochemical parameters for each nucleant layer used

**Nucleant layer**	**Potentiostatic**		**Galvanostatic**		**Pulsed current**			
	***E *(V)**	**Time (s)**	***I *(mA)**	**Time (s)**	***I *(mA)**	***t***_**ON **_**(s)**	***t***_**OFF **_**(s)**	**Time (s)**
ITO	−1	600	−4	600	−4	1	1	1,200
Spin-coated ZnO	−1	600	−1.75	600	−1.75	1	1	1,200
Sputtered ZnO	−0.8	600	−1.5	600	−1.5	1	1	1,200

## Results and discussion

### Scanning electron microscopy and atomic force microscopy

The morphological and structural ZnO nanorod properties for each different substrate were analyzed by SEM (JSM-6300, Jeol scanning electron microscope, JEOL, Tokyo, Japan) operating at 20 kV and AFM (Veeco Multimode, Veeco Instruments Inc., Plainview, NY, USA).

**Figure 2:**
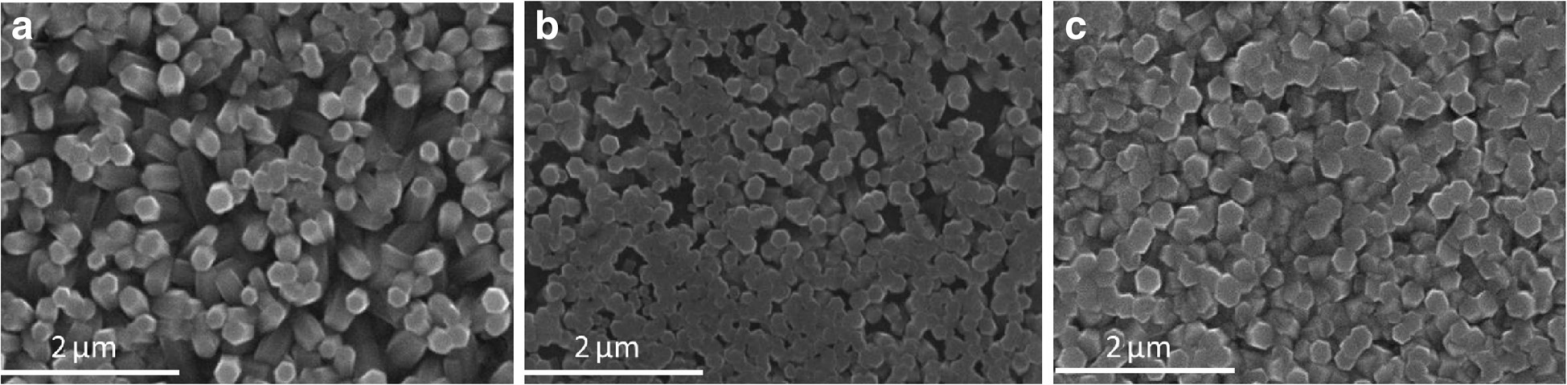
**SEM of ZnO nanorods obtained by electrodeposition method on ITO substrate.** Via (**a**) Potentiostatic, (**b**) galvanostatic, and (**c**) pulsed-current methods.

Figure 2 shows the ZnO nanorods obtained on ITO substrates under the three different electrochemistry processes: potentiostatic, galvanostatic, and pulsed-current methods. It can be seen that the nanostructure density and alignment with pulsed-current process improved and that the nanostructure becomes a continuous layer. When pulsed current is applied on a substrate without a previous ZnO nucleant layer, the nucleus of ZnO is homogeneously formed along the whole surface [13]. The average diameter obtained in this case is 220 nm.

**Figure 3:**
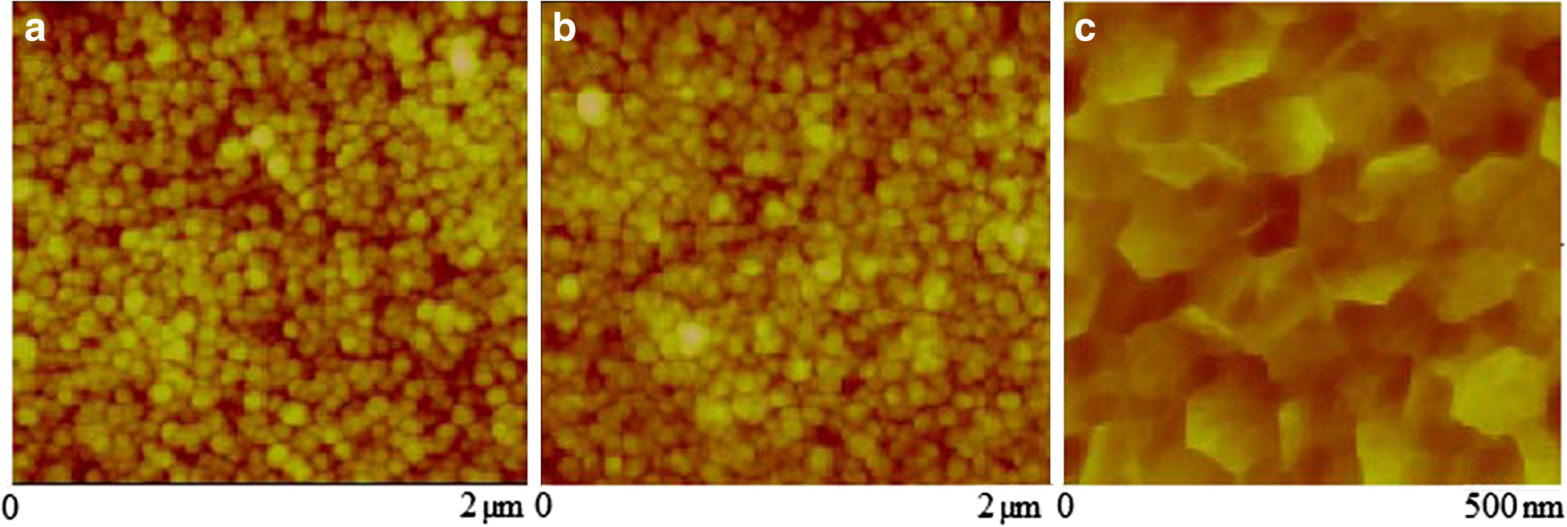
**AFM of ZnO nanorods obtained by electrodeposition method on ZnO spin-coated substrate.** Via (**a**) potentiostatic, (**b**) galvanostatic, and (**c**) pulsed current.

For the substrates with spin-coated ZnO as nucleant layer, it is necessary to analyze the nanostructures with AFM due to the low roughness of the sample (Ra = 4 nm). In Figure 3, the nanorods obtained by potentiostatic, galvanostatic, and pulsed-current methods are shown. In the case of applying a pulsed current, the nanostructure morphology results are more defined, with a lower diameter than the ITO substrate case, around 100 nm of average diameter. The substrate obtained by spin-coating process generates a homogeneous layer across the surface, with very low roughness [21] and small grains of material, so the current applied to the surface is distributed homogenously.

**Figure 4:**
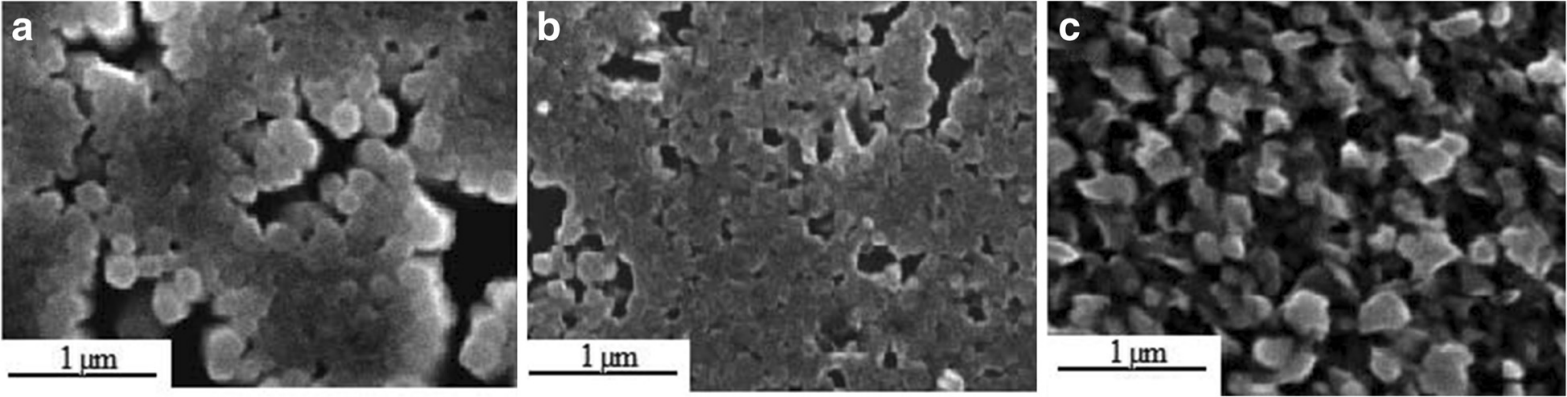
**SEM of ZnO nanorods obtained by electrodeposition method on ZnO sputtered substrate.** Via (**a**) potentiostatic, (**b**) galvanostatic, and (**c**) pulsed current.

For the ZnO sputtered nucleant layer substrate, the result is quite different. Figure 4 shows the SEM images for the three electrodeposition processes done. In this case, the pulsed-current process yields the worst obtained morphology in comparison with ITO and spin-coated substrates. The sputtering process generates a heterogeneous layer on the surface. This is due to a small variation of thickness along the surface due to the system geometry imposed on the equipment, generating poor uniformity of the applied current. Thus, a better nanostructure is obtained through the potentiostatic electrodeposition process, yielding an average nanorod diameter of 220 nm, like the one obtained for ITO.

### Optical characterization

**Figure 5:**
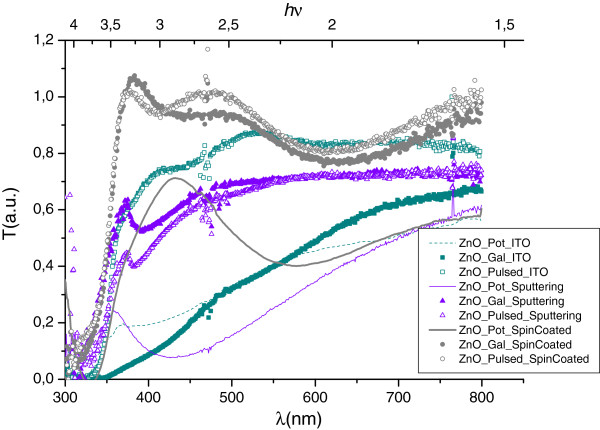
**Transmission spectra.** For ZnO nanorod growth by galvanostatic and pulsed-current electrodeposition on ITO, sputtered ZnO, and spin-coated ZnO as substrate.

Optical transmission characteristics were also realized at room temperature with a Newport UV–VIS spectrophotometer (Irvine, CA, USA) in the 300- to 850-nm wavelength range. The results for the galvanostatic and pulsed-current electrodeposition samples are show in Figure 5.

As shown in Figure 5, the transmission behavior is strongly dependent on the substrate used in the electrodeposition process, with all of them being transparent at wavelength above 350 nm. Spin-coated and sputtered substrates show similar features on the transmission signal for the galvanostatic and pulsed-current processes used. On the contrary, both processes have a significant difference on ITO substrate, with the one obtained by pulsed current having better transmission.

The ZnO obtained revealed a poor crystalline nanostructure when the potentiostatic growth method was applied for the three substrates used. This effect can be seen in the optical behavior of the transmission curves where the optical bandgap is not clearly defined due to electronic defects inside the structure. The best optical result is for the spin-coated substrate, in agreement with the AFM analysis (Figure 3), which shows a homogeneous nanostructure.

### Optical bandgap

**Figure 6:**
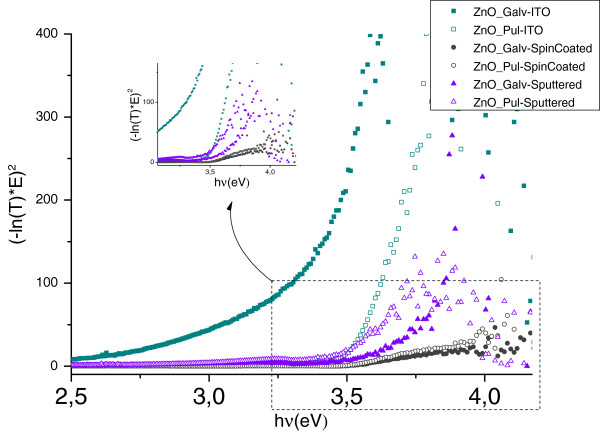
**Optical bandgap of ZnO nanowire array.** Plot of (−ln*T* × *h*ν)^2^ vs photon energy of ZnO nanowire array growth by galvanostatic and pulsed-current electrodeposition on ITO, sputtered ZnO, and spin-coated ZnO as substrate.

Optical bandgap of ZnO has been reported from 3.27 eV for the single crystal to 3.55 eV for the electrodeposited films [21,22]. The electrodeposited ZnO films or nanostructures exhibit bandgap between 3.3 and 3.55 eV, depending on the structural morphologies and crystal defects. Assuming an absorption coefficient α∝−ln*T (T* is transmittance) corresponding to a direct bandgap of ZnO, [23] the bandgap of the ZnO nanowires is estimated from the linear fit in the plot of (−ln*T* × *h*ν)^2^ against the energy *h*ν, as shown in Figure 6 and Table 2 for each sample. Analysis is not presented for potentiostatic samples because the absorption band edge is not sufficiently well defined to be considered for the linear fit, as was described in the optical characterization.

**Table 2:** Optical bandgap for ZnO nanorods obtained by electrodeposition on different substrates

**Sample**	**Eg (eV)**
Pulsed current on ITO	3.51
Galvanostatic on ITO	3.33
Pulsed current on spin-coated ZnO	3.51
Galvanostatic on spin-coated ZnO	3.51
Pulsed current on sputtered ZnO	3.46
Galvanostatic on sputtered ZnO	3.56

The optical bandgap for all samples obtained is in agreement with the theoretical ZnO bandgap [24], although the results show that galvanostatic electrodeposition on ITO substrate is quite different from the other ones, which was expected from microstructure analysis.

## Conclusions

In the present work, the influence of the nucleant layer on the process of vertically aligned ZnO nanowires grown using electrochemical reactions has been described and analyzed. It can be concluded that the nucleant layer has a crucial role in the morphological, structural, and optical properties of the electrodeposited material. In this sense, the spin-coated substrate has demonstrated to be the more easily controlled in order to obtain optimal electrodeposited nanostructures.

## Competing interests

The authors declare that they have no competing interests.

## Authors’ contributions

MDRT carried out the electrodeposition process, sputtering and characterization techniques, and the study of the results, and drafted manuscript. HB contributed to the spin-coated experimental section. LCD, MAHF, and HJB conceived of the study, participated in its design and coordination, and helped draft the manuscript. All authors read and approved the final manuscript.
